# Propofol inhibits neuroinflammation and metabolic reprogramming in microglia *in vitro* and *in vivo*

**DOI:** 10.3389/fphar.2023.1161810

**Published:** 2023-06-13

**Authors:** Shuyuan Guan, Lingbin Sun, Xihua Wang, Xirui Huang, Tao Luo

**Affiliations:** Department of Anesthesiology, Peking University Shenzhen Hospital, Shenzhen, China

**Keywords:** Microglia, Propofol, neuroinflammation, metabolic reprogramming, HIF-1α

## Abstract

Microglial activation-induced neuroinflammation is closely related to the development of sepsis-associated encephalopathy. Accumulating evidence suggests that changes in the metabolic profile of microglia is crucial for their response to inflammation. Propofol is widely used for sedation in mechanically ventilated patients with sepsis. Here, we investigate the effect of propofol on lipopolysaccharide-induced neuroinflammation, neuronal injuries, microglia metabolic reprogramming as well as the underlying molecular mechanisms. The neuroprotective effects of propofol (80 mg/kg) *in vivo* were measured in the lipopolysaccharide (2 mg/kg)-induced sepsis in mice through behavioral tests, Western blot analysis and immunofluorescent staining. The anti-inflammatory effects of propofol (50 μM) in microglial cell cultures under lipopolysaccharide (10 ng/ml) challenge were examined with Seahorse XF Glycolysis Stress test, ROS assay, Western blot, and immunofluorescent staining. We showed that propofol treatment reduced microglia activation and neuroinflammation, inhibited neuronal apoptosis and improved lipopolysaccharide-induced cognitive dysfunction. Propofol also attenuated lipopolysaccharide-stimulated increases of inducible nitric oxide synthase, nitric oxide, tumor necrosis factor-α, interlukin-1β and COX-2 in cultured BV-2 cells. Propofol-treated microglia showed a remarkable suppression of lipopolysaccharide-induced HIF-1α, PFKFB3, HK2 expression and along with downregulation of the ROS/PI3K/Akt/mTOR signaling pathway. Moreover, propofol attenuated the enhancement of mitochondrial respiration and glycolysis induced by lipopolysaccharide. Together, our data suggest that propofol attenuated inflammatory response by inhibiting metabolic reprogramming, at least in part, through downregulation of the ROS/PI3K/Akt/mTOR/HIF-1α signaling pathway.

## 1 Introduction

Sepsis is a life-threatening organ dysfunction caused by a dysregulated host response to infection. It is estimated that the incidence of sepsis around the world is up to 50 million cases and approximately 5.3 million deaths annually (Karampela et al., 2019). Neuroinflammation and neuronal death can be heavily caused by sepsis (Gao et al., 2011; Ransohoff, 2016; Joers et al., 2017; Hickman et al., 2018); these can even be induced by a single small dose of lipopolysaccharide administration reported previously (Mai et al., 2017; Sun et al., 2019).

Microglia, as the primary innate immune cells in the central nervous system, are the central pathogeneses of neuroinflammation (Shemer et al., 2015). Following inflammatory stimuli, microglia were activated and subsequently changed morphology and released inflammatory cytokines, chemokines and reactive oxygen species (ROS) (Zhang et al., 2009; Vana, et al., 2011); all of which in turn induced neuronal injuries or death (Chhor et al., 2013). Thus, inhibition of microglia over activation has been considered to be an effective treatment of neuroinflammation-related diseases. Recent studies suggest that microglia can change their phenotype and metabolic state in response to immune challenge (Lauro et al., 2019; Macedo et al., 2019). For example, during the classic inflammatory activation process, cellular metabolism is reprogrammed from oxidative phosphorylation (OXPHOS) to aerobic glycolysis, a phenomenon known as the Warburg effect (Schuster et al., 2015; Nair et al., 2019). Pharmacologic inhibition of glycolysis blunted the M1 polarization in microglia (Bejoy et al., 2019).

Propofol is a potent intravenous hypnotic agent that is widely used for general anesthesia induction and maintenance, as well as for ICU sedation. Previous studies demonstrated that propofol has immunomodulatory and antioxidative properties. For instance, propofol was reported to reduce endotoxin-induced inflammatory responses in septic rats (Yang et al., 2018). Also, propofol was found to decrease proinflammatory cytokine and iNOS production from lipopolysaccharide-stimulated human monocytic THP-1 cells (Nie et al., 2015). We previously reported that propofol induced neuroprotection is associated with inhibiting microglia activation (Luo et al., 2013; Luo et al., 2015; Zheng et al., 2017). However, it remains to elucidate whether and how propofol affect microglial metabolic state in response to insult, e.g., lipopolysaccharide.

In the present study, we evaluated the therapeutic effects of propofol on neuroinflammation, cell injury and cognitive dysfunction associated with lipopolysaccharide induced sepsis and the underlying mechanisms in both *in vivo* and *in vitro* settings.

## 2 Materials and methods

### 2.1 Animals and drug administration

C57/BL6 male mice (8–12 weeks old), purchased from GemPharmatech Co. Ltd. (Guangdong, China), were housed at a temperature of 22°C ± 1°C and relative humidity 50%–60% under a 12-h/12-h light–dark cycle, with free access to food and water. The experimental protocol was compliance with Local Animal Care and Use Committee and the Guideline for the Care and Use of Laboratory Animals. Mice were intraperitoneally challanged with 2 mg/kg lipopolysaccharide (L2630, Sigma-Aldrich), dissolved and diluted in sterile normal saline. Half an hour before lipopolysaccharide administration, propofol was injected intraperitoneally at a dose of 80 mg/kg. The median effective dose (ED50) of propofol to induce loss of righting reflex is about 70 mg/kg (Nguyen et al., 2009). Under 80 mg/kg, all the experimental animals were fully sedated.

### 2.2 Y-maze

The spatial working memory was tested using a Y-maze (SA204, SANS, China). The Y-maze consisted of three identical beige plastic arms at 120° angle that were labeled A, B, C. Each arm was 10 cm in width, 40 cm in length, and 20 cm in height. The mice were placed individually in the far end of one arm, and their movement was monitored by a webcam and analyzed using the Any-Maze software (Stoelting, Illinois, 10 United States). and the sequence (i.e., ACABC, etc.) and number of arm entries were recorded manually for each mouse for 8 min. A mouse was considered to have entered an arm if its whole body (except for the tail) entered the arm and to have exited if the whole body exited the arm. If an animal entered three different arms on consecutive choices (i.e., ABC, CAB, or BAC but not ABA), it was counted as a spontaneous alternation performance (SAP), which is an indication of sound working memory. If an animal went from A to B and came back to B, it was considered as an alternate arm return (AAR). Any time a mouse went from arm to the center area and came back to the same arm, it was counted as the same arm return (SAR). The score of alternation was calculated using the formula: Score = [number of alternating triads/(total number of triads minus 2)] × 100% (Zhong et al., 2019). The total number of arm entries was measured as an index of locomotor activity to rule out the interference of changes in motility with the parameters of learning and memory (Mohammadi et al., 2016).

### 2.3 BV2 microglial cell culture and treatment

BV2 cell lines were cultured and maintained in DMEM with 10% FBS, 100 U/mL penicillin, and 100 mg/mL streptomycin at 37°C in a humidified incubator with 5% CO_2_. Previous studies from our and other groups have found that propofol at concentrations between 12.5 and 200 μM dose dependently attenuates inflammatory response in lipopolysaccharide-activated microglia *in vitro* (Zheng et al., 2017). The blood plasma concentrations of propofol are reportedly 10–60 μM at anesthesia induction and maintenance (Murphy, et al., 1996; Adachi et al., 2005). Therefore, BV2 cells were treated with or without lipopolysaccharide (10 ng/mL), in the presence or absence of propofol (50 μM). For cellular signaling pathway study, BV2 cultures were pretreated with CoCl_2_ (100 μM), Kc7f2 (10 μM), Insulin (100 nM), Dimethyloxallyl Glycine (DMOG) (100 μM) or LY294002 (10 μM), respectively (Huang et al., 2016), followed by lipopolysaccharide (10 ng/mL) and/or propofol (50 μM) treatment accordingly.

### 2.4 Immunocytochemistry and immunohistochemistry

For *in vivo* experiment, 4 h after intraperitoneal injection with lipopolysaccharide and propofol, mice were sacrificed under deep anaesthesia and transcardially perfused with 0.9% saline solution, followed by 4% PFA in 0.1 M PBS. The brains were removed and immersed in 4% PFA for 24 h, and then cryoprotected in 30% sucrose solution in PBS (pH 7.4). Subsequently, serial coronal sections were then prepared using a microtome. For *in vitro* study, BV2 microglia cells were fixed with 4% Paraformaldehyde (PFA) for 15 min at 37°C. After fixation, slides were washed in PBS, blocked with 5% Bovine albumin and 0.5% Triton X-100 in PBS, and then incubated with primary antibodies (mouse anti-Iba-1: 1:200, NCNP24, Wako, Japan; mouse anti-NeuN: 1:200, ab104224; rabbit anti mouse PFKFB3: 1:200, ab181861; rabbit anti mouse PSD-95: 1:200; ab18258, Abcam, United States) at 4°C overnight followed by secondary antibodies (Alexa Fluor 594-labeled goat anti-mouse IgG: 1:500, ab150116 or Alexa Fluor 488-labeled goat anti-rabbit IgG: 1:500, ab150077; Abcam, United States) as appropriate. Counterstaining was then performed with DAPI (1 ug/mL). The slides were examined under a Confocal Laser Scanning Microscope (Leica, Solms, Germany).

### 2.5 Measurement of nitrite production

BV2 microglial cells were cultured in 96-well plates and then propofol at 50 μM concentration plus 10 ng/mL lipopolysaccharide were given for 24 h. The cell supernatants were collected and mixed with Griess reagent (Beyotime) and the nitrite production was measured with a microplate reader at an absorbance of 540 nm according to the manufacturer’s instruction.

### 2.6 Western blot analysis

The hippocampus or cultured cells pellet were homogenized in RIPA Lysis Buffer (Beyotime, China) containing protease inhibitor PMSF (Beyotime, China). The supernatants were collected and their protein concentrations were measured with BCA protein analysis kit (Biosharp, BL521A). Total proteins of equivalent amounts were separated in 12% SDS-PAGE and then transferred to polyvinylidene fluoride (PVDF) membranes (MILLIPORE, United States). After blocked with 5% skim milk in buffer, the membranes were incubated overnight at 4°C with the corresponding primary anti-bodies: anti-iNOS (A18247), anti-HIF-1α (A16873), anti-HK2 (A0994), anti-COX-2 (A1253), anti-β-actin (AC026) (all from Abclonal, Wuhan, China); Anti-IL-1β (ab9277), anti-PFKFB3 (ab181861) and anti-TNF-α (ab6671) (all from Abcam, Cambridge, United States); Anti-phospho-PI3K antibody (#4228), anti-PI3K antibody (#4257), anti-phospho-Akt (#4060), anti-Akt antibody (#4691), anti-phospho-mTOR (#5536), anti-mTOR (#2983) (all from Cell Signaling Technology, Trask Lane, United States). They were then incubated with horse-radish peroxidase–conjugated secondary antibodies at 25°C for 2 h. After washes with TBST, the protein bands were visulazed with chemiluminescence detection system (Tanon 5200) and analyzed by ImageJ (NIH, Bethesda, MD, United States).

### 2.7 Detection of intracellular ROS

Intracellular ROS was detected by means of an oxidation-sensitive fluorescent probe (DCFH-DA). After treated with lipopolysaccharide and/or propofol, the cultured cells were then washed twice with cold PBS and incubated with DCFH-DA at room temperature for 30 min in dark. Fluorescent signals were detected with a fluorescence microscopy (Leica, Solms, Germany). The fluorescence intensity of ROS level was quantified with ImageJ.

### 2.8 Extracellular acidification rate

BV2 cells were seeded in XFe 96-well microplates (Agilent Technologies, Sana Clara, United States) for 12 h, followed by lipopolysaccharide and/or popofol treated for a further 9 h. Cells were then washed and incubated in basal medium (Agilent Technologies) at 37°C for 45 min. Extracellular acidification rate was measured in real-time with Glycolysis Stress Test Kit (Santa Clara, CA, United States) using the Seahorse XFe96 Analyser (Agilent Technologies) following manufacturer’s instructions.

### 2.9 Statistical analyses

Data are expressed as means ± SD and analysed with one-way ANOVA followed by Tukey’s *post hoc* for statistical comparisons (Prism 6.0, GraphPad Software, United States). A statistical significance was set at the level of *p* less than 0.05.

## 3 Results

### 3.1 Propofol reduces neuronal injury and improves cognitive recovery in septic mice

**FIGURE 1:**
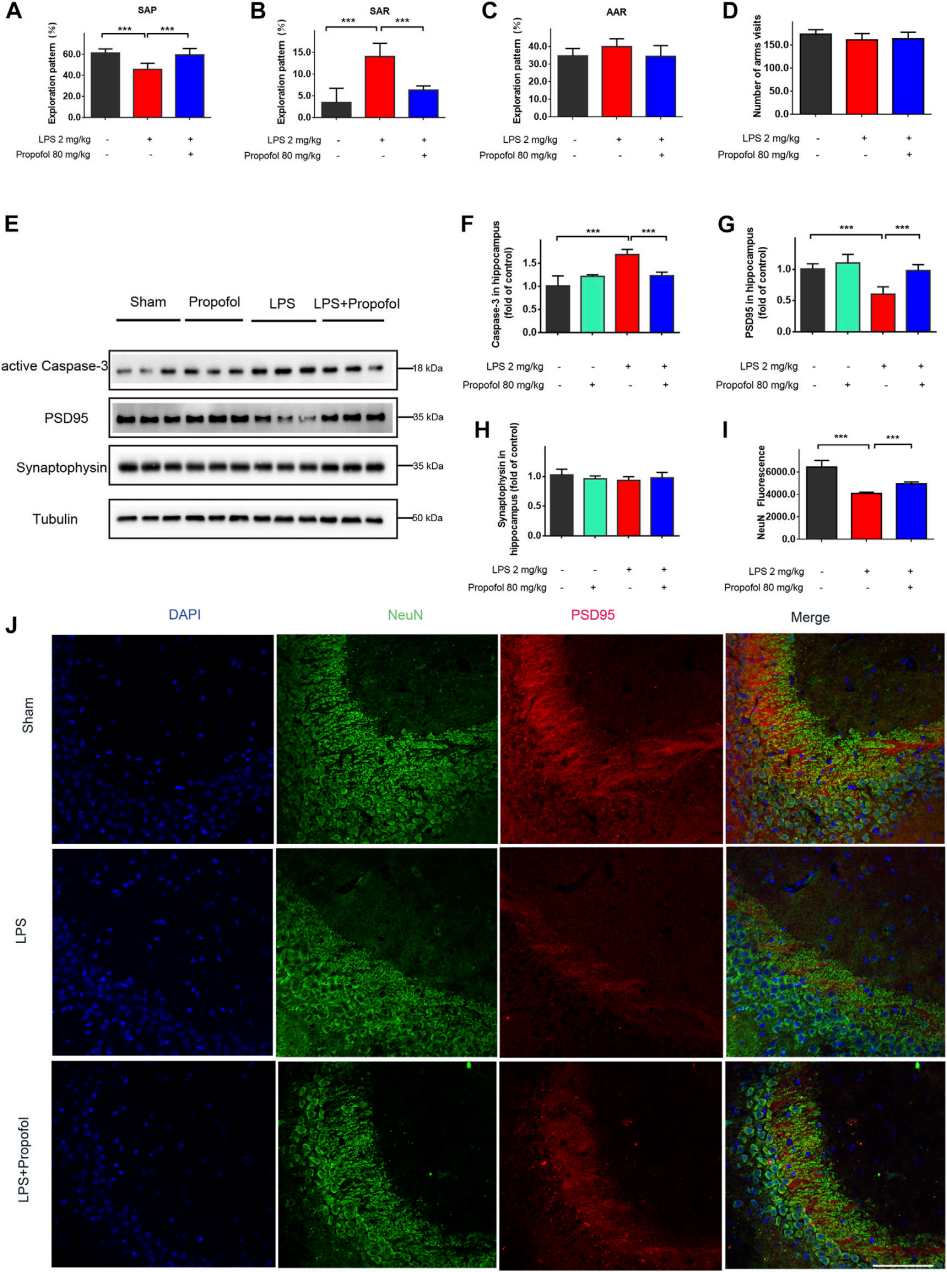
Propofol inhibits lipopolysaccharide-induced hippocampal injury and cognitive deficit *in vivo*. The animals were subjected to Y-maze to test spatial working memory at 24 h after Intraperitoneal injection of lipopolysaccharide. **(A–D)** Propofol improved SAP, and reduced SAR. SAP is defined as the percentage of triads that an animal goes into three different arms of the Y-maze in a triad entry; AAR as the percentage of an animal goes into alternative arms in a triad entry, and SAR as the percentage of an animal returns to the same arm in any consecutive entries in a triad entry. **(E–H)** Western blot analysis showed that propofol significant decreased the expression of active Caspase-3, PSD95 at 4 hours after intraperitoneal injection of lipopolysaccharide. **(I, J)** The Immunofluorescence staining (scale bar below: 25 μm) showed DAPI (blue), PSD95 (red), colocalized (merged) with NeuN (green). The data are expressed as means ± SD (n = 6-8 animals); ***p < 0.001 for comparisons as shown.

Systemic administration of lipopolysaccharide has been known to cause learning and memory defects (O'Neill et al., 2021). We examined the effect of propofol on spatial working memory function in lipopolysaccharide injected mice by the Y-maze. Compared with control mice, lipopolysaccharide treatment significantly reduced the probability of spontaneous alternating behavior (SAP). Propofol administration prevented the decline of SAP induced by lipopolysaccharide (Figure 1A). Lipopolysaccharide increased the same arm return probability (SAR), which was reversed by propofol (Figure 1B). Alternate arm return (AAR) showed no difference among the groups (Figure 1C). Moreover, the general locomotor activity, measured as the total number of arm entries, was not affected by lipopolysaccharide or propofol when compared with saline group (Figure 1D).

The hippocampus neuronal injury contributes to impaired cognitive and memory function (Lee et al., 2008). To determine whether propofol may affect neuronal survival, neuronal cell apoptosis was determined by caspase-3 expression in the hippocampus. Compared to the control group, the cleaved caspase-3 was increased in the lipopolysaccharide-challenged mice, which was decreased by propofol administration (Figures 1E, F). In line with caspase-3 expression, NeuN expression confirmed that propofol protected the animals from lipopolysaccharide induced neuronal cell death (Figures 1E, I, J). We next determined the postsynaptic protein PSD-95 and the presynaptic protein synaptophysin expression. The protein level of PSD-95 were significantly decreased after lipopolysaccharide treatment. While propofol attenuated lipopolysaccharide-induced downregulation of PSD-95 (Figures 1E, G, J). The synaptophysin expression did not reach statistical significance by either lipopolysaccharide or propofol treatments at the time point in our experiment (Figures 1E, H).

### 3.2 Propofol attenuates lipopolysaccharide-induced hippocampal inflammation in septic mice

**FIGURE 2:**
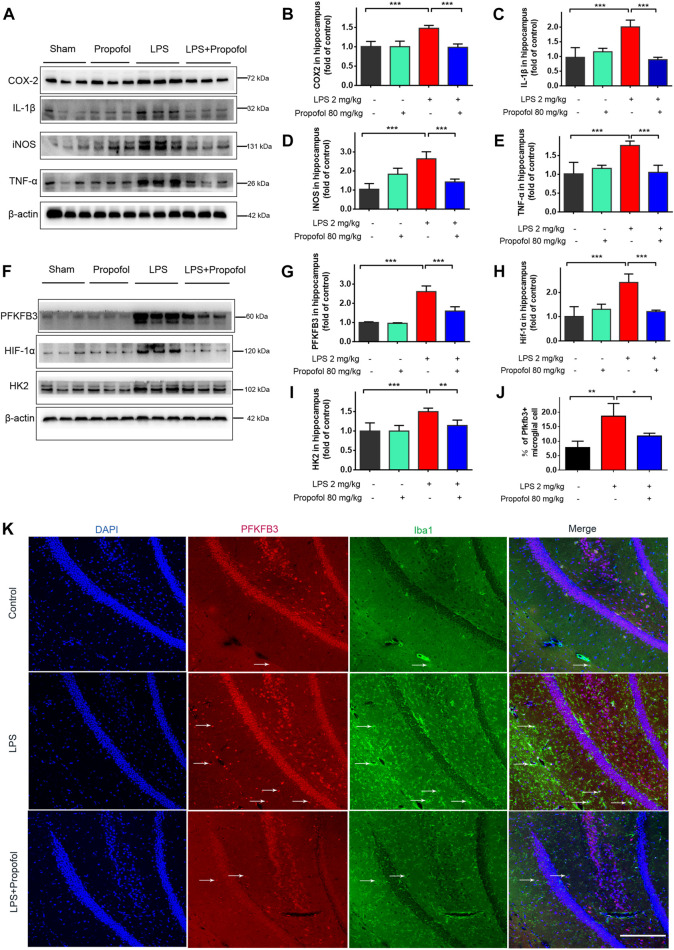
Propofol attenuates lipopolysaccharide-induced hippocampal HIF-1α, glycolytic enzyme expressions and proinflammatory mediators in mice. Mice of 8–12 week old were randomly—assigned to control, propofol, lipopolysaccharide (LPS), LPS + propofol. Four hours after lipopolysaccharide intraperitoneal injection, the mice were euthanized. **(A–I)** Their hippocampus was harvested and assayed by Western blot for HIF-1α, HK2, PFKFB3, IL-1β, COX2, iNOS and TNF-α. **(J, K)** The Immunofluorescent staining (scale bar 100 μm) showed DAPI (blue), PFKFB3 (red), colocalized (merged) with Iba1–positive reactive microglial (green). Data are expressed as means ± SD (n = 6 animals); *p < 0.05,**p < 0.01; ***p < 0.001 for comparisons as shown.

The inflammatory response in the hippocampus was evaluated by Western blot analysis. The inflammatory markers of COX-2、IL-1β、iNOS and TNF-α expression were all increased 4 h after lipopolysaccharide challenge, and propofol significantly decreased these elevations (Figures 2A–E). Propofol administration downregulated lipopolysaccharide-induced hippocampal HIF-1α, PFKFB3 and HK2 expression (Figures 2F–I). Furthermore, fluorescence staining confirmed consistently that the amount of the glycolysis maker PFKFB3 expression in Iba-1-positive cells was increased in lipopolysaccharide challenged animal brain. Such increases were less pronounced in the propofol treated mice (Figures 2J, K).

### 3.3 Propofol decreases lipopolysaccharide-induced inflammatory mediator productions in microglial cells

**FIGURE 3:**
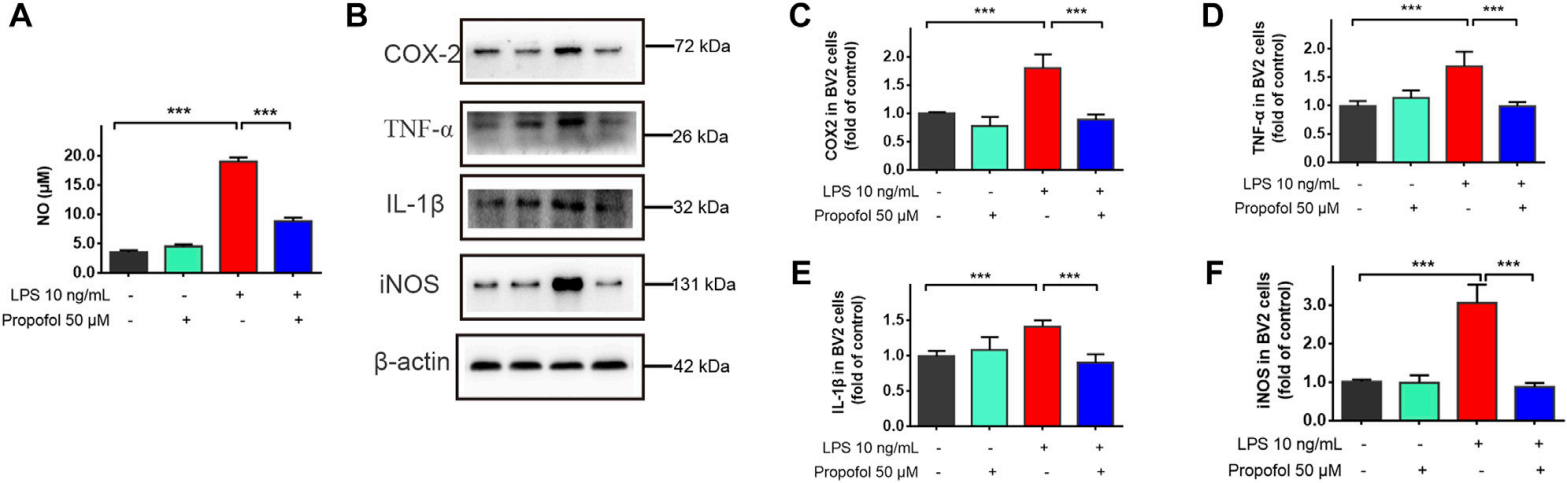
Propofol downregulates lipopolysaccharide-stimulated production of proinflammatory mediators in microglial cells. BV2 microglial cells were treated with lipopolysaccharide (10 ng/mL) and propofol (50 μM). **(A)** At 24 h of treatment, the production of a nitric oxide was measured by Griess assay. **(B–F)** At 9 h of treatment, the expression of COX-2, TNF-α, IL-1β and iNOS was measured by Western blot. Data are means ± SD (n = 6 independent measurements); ***p < 0.001 for comparisons shown.

To further investigate the neuroprotective mechanism of propofol against lipopolysaccharide-induced neurotoxicity, immortalized murine microglia BV-2 cells were co-incubated with propofol (50 μM) and/or lipopolysaccharide (10 ng/ml). Nitric oxide, COX-2, TNF-α, IL-1β and iNOS production were all significantly increased after lipopolysaccharide stimulation compared with the untreated controls. These upregulation of inflammatory mediators were inhibited by propofol treatment (Figures 3A–F).

### 3.4 Propofol regulates lipopolysaccharide-induced enhancement of mitochondrial and glycolytic function *in vitro*

**FIGURE 4:**
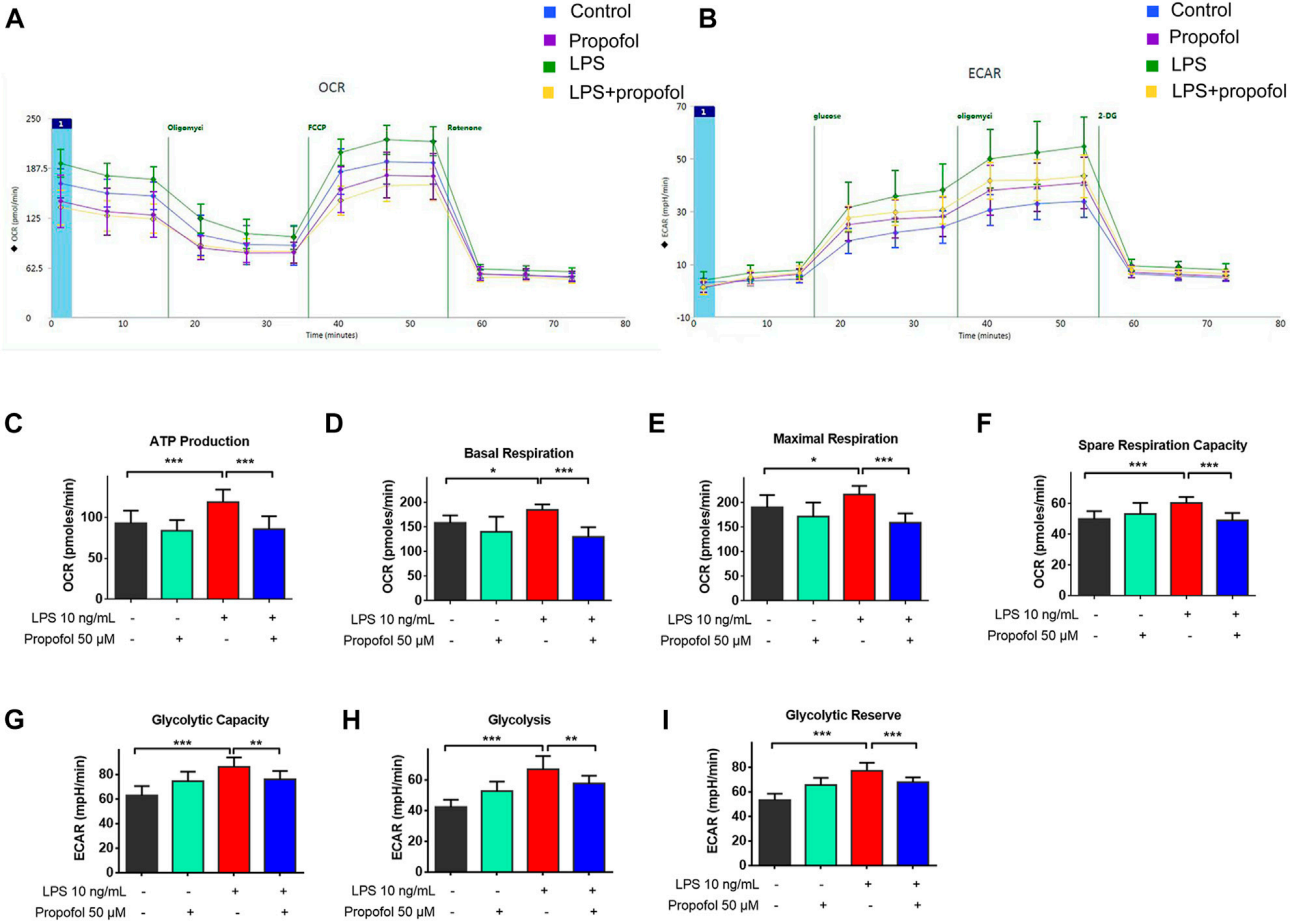
Propofol regulates mitochondrial and glycolytic function in microglia. BV2 microglial cells were treated with lipopolysaccharide (10 ng/mL) and propofol (50 μM) for 9 h **(A–I)** The dynamic changes of glycolysis and TCA cycle were measured by ECAR and OCR, respectively, using a Seahorse extracellular flux analyzer. Data are means ± SD (n = 12 independent measurements); *p < 0.05, **p < 0.01, ***p < 0.001 for comparisons shown.

Microglial activation triggers metabolic reprogramming with increasing glucose uptake, which was accompanied by a reduction in oxidative phosphorylation and an increase in glycolysis (Gimeno-Bayón et al., 2014; Nair et al., 2019). The oxygen consumption rate (OCR) and extracellular acidification rate (ECAR) were measured by the Seahorse XFe96 as indicators of mitochondrial respiration and glycolysis. As shown in Figures 4A–F, ATP-linked OCR, basal OCR, FCCP-induced maximal OCR and spare respiratory capacity (SRC) were increased following exposure to lipopolysaccharide compared with controls. The glycolysis, glycolytic capacity and glycolytic reserve were also significantly increased (Figures 4G–I). Such effects of lipopolysaccharide were significantly attenuated by propofol (Figures 4A–I).

### 3.5 HIF-1α is required for the anti-inflammatory effect of propofol

**FIGURE 5:**
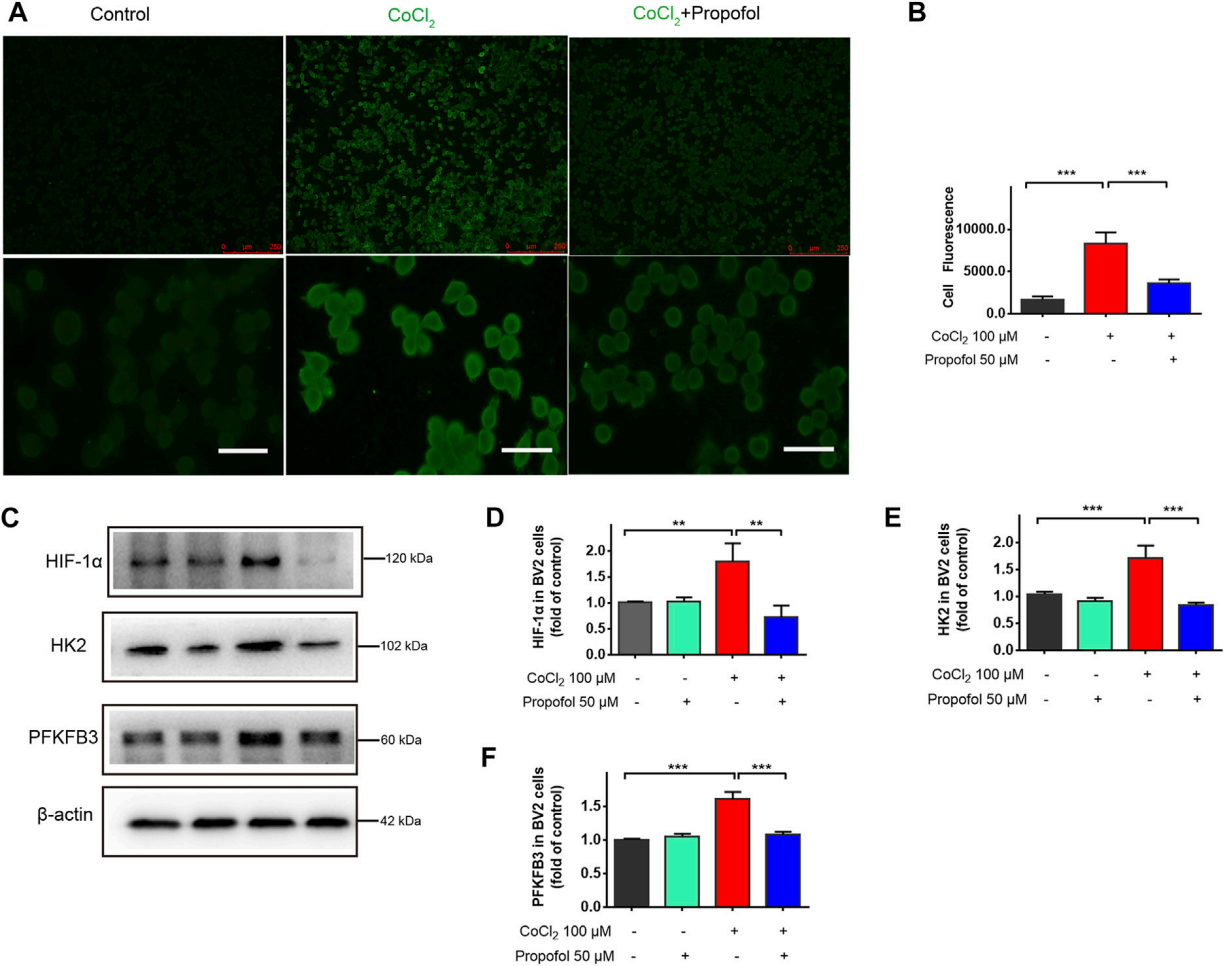
Propofol inhibits HIF-1α and glycolytic enzymes expression in CoCl_2_-treated microglia. BV2 cells were pretreated with 100 μM CoCl_2_ for 24 h and then treated with 50 μM propofol for 9 h. **(A)** The fluorescent micrographs of HIF-1α expression (scale bar below: 25 μm). **(B)** The fluorescence intensity of HIF-1 α staining qualified with ImageJ. **(C–F)** The expression of HIF-1α, HK2 and PFKFB3 by Western blot. Data are expressed as means ± SD (n = 3 independent measurements); **p < 0.01; ***p < 0.001 for comparisons as shown.

HIF-1α plays a crucial role in regulating cell metabolic reprogramming. To explore whether HIF-1α mediated metabolic reprogramming is involved in the protection afforded by propofol, HIF-1α activation in BV2 cells were stimulated by CoCl_2_ (100 μM) for 24 h followed by co-administration with propofol for additional 9 h. The protein level of HIF-1α, HK2 and PFKFB3 in microglia were significantly increased after CoCl_2_ treatment, while these up-regulations with CoCl_2_ were mitigated by propofol (Figures 5C–F). Similarly, CoCl_2_ caused accumulation of HIF-1α signal in fluorescence through inhibiting HIF-1α degradation. Such effects were less pronounced when treated with propofol (Figures 5A, B).

**FIGURE 6:**
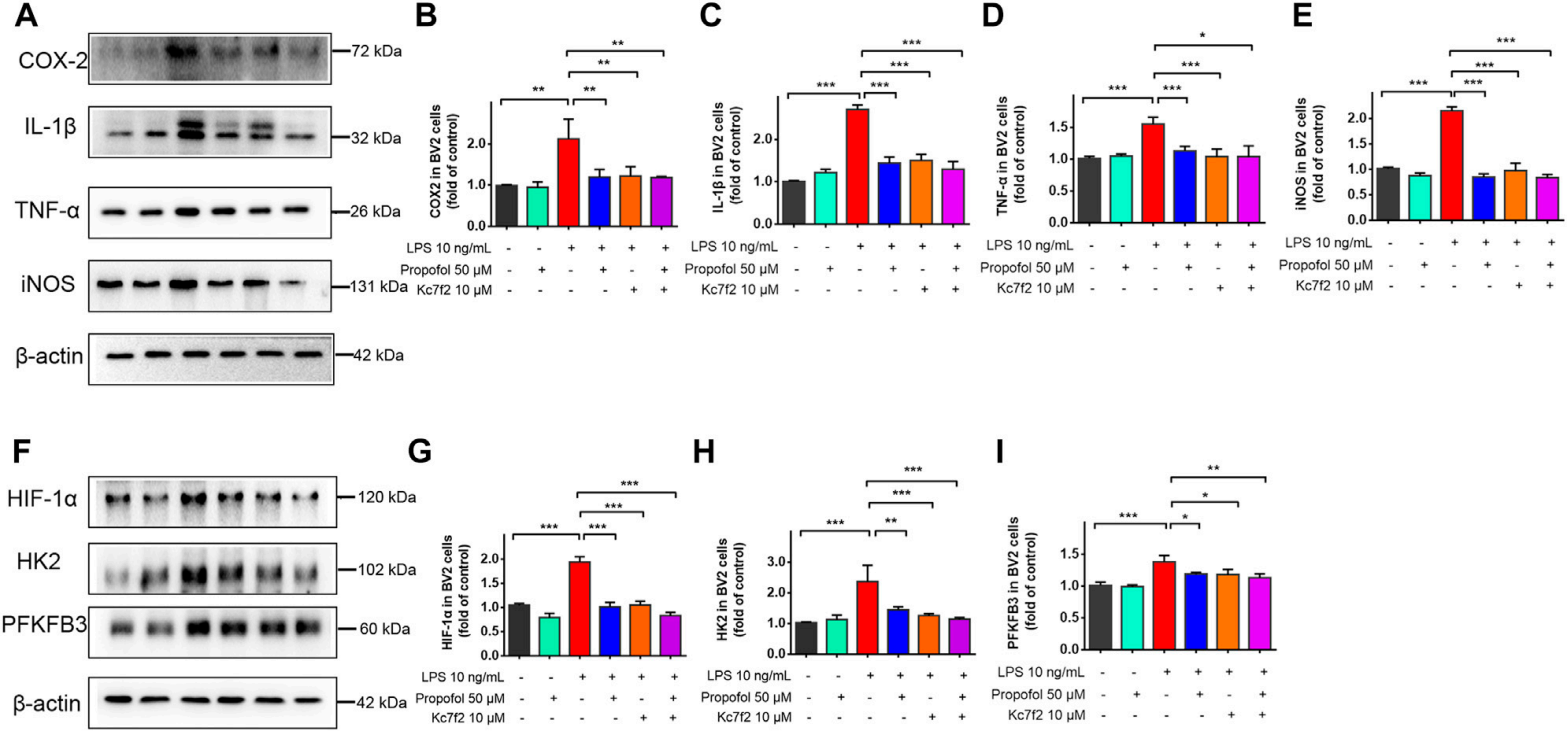
The inhibitory effect of propofol on microglial activation via HIF-1α-glycolysis. Six groups of randomly assigned cells were treated with medium vehicle (control), propofol, lipopolysaccharide (LPS), LPS + propofol, LPS + Kc7f2, Kc7f2. **(A–I)** The expression of COX-2, TNF-α, IL-1β, iNOS, HIF-1α, HK2 and PFKFB3 by Western blot. Data are expressed as means ± SD (n = 3 independent measurements); *p < 0.05; **p < 0.01; ***p < 0.001 for comparisons shown.

BV2 cells were further treated with HIF-1α inhibitor Kc7f2 (10 μM) for 24 h, and then exposed to lipopolysaccharide and propofol for 9 h. The inhibitory effect of propofol on lipopolysaccharide-induced neuroinflammation was no longer exist after downregulation of HIF-1α by Kc2f7 (Figures 6A–E). Similarly, propofol had no further effect on HIF-1α, HK2 and PFKFB3 expression when Kc7f2 was administered to BV2 cells (Figures 6F–I).

### 3.6 Propofol inhibits microglial activation through ROS/PI3K/Akt/mTOR/HIF-1α pathway

**FIGURE 7:**
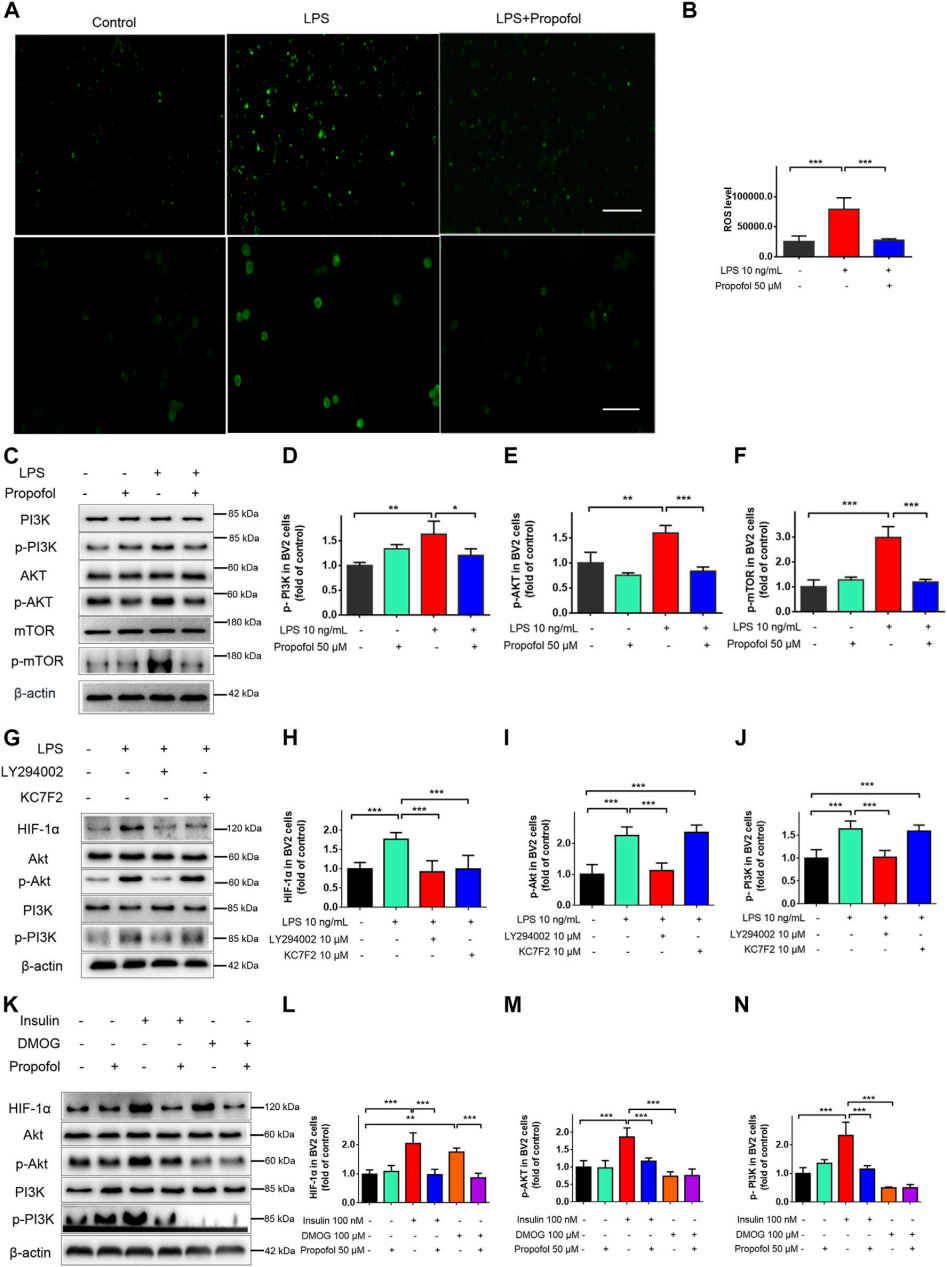
Propofol regulates HIF-1α-mediated metabolic reprogramming through inhibiting ROS/PI3K/Akt/mTOR signaling pathway in microglia. **(A, B)** The ROS fluorescence (scale bar below: 50 μm). **(C–F)** The expression of PI3K, Akt and mTOR after 9 h of treatment with lipopolysaccharide (LPS) (10 ng/mL) and/or propofol (50 μM) (n = 3 independent measurements). And then BV-2 cells were pretreated with LY294002, Kc2f7, DMOG, or Insulin, followed by treatment with lipopolysaccharide or propofol for 9 h **(G–N)** The expression of PI3K, p-PI3K, Akt, p-Akt and HIF-1α. Data are expressed as means ± SD (n = 4 independent measurements); *p < 0.05, **p < 0.01, ***p < 0.001 for comparisons shown.

The ROS/PI3K/Akt/mTOR signaling pathway is known to play a critical regulatory role in microglial injury during hypoxia (Chen et al., 2016). Next, we examined the involvement of the ROS/PI3K/Akt/mTOR/HIF-1a pathway in propofol anti-inflamamtion in BV2 cells. As shown in Figures 7A, B, the fluorescent intensity of ROS was significantly increased in lipopolysaccharide treated cells, whereas propofol inhibits ROS production. In addition, the phosphorylation of PI3K, Akt and mTOR was significantly increased after being treated with lipopolysaccharide and these up-regulations were mitigated by propofol (Figures 7C–F).

LY294002 is a typical PI3K inhibitor, and Kc2f7 is a specific HIF-1α inhibitor. To further study the upstream and downstream relationship between PI3K/Akt and HIF-1α. BV2 cells were pretreated with LY294002 or Kc2f7 followed by lipopolysaccharide for 9 h. PI3K/Akt pathway was activated by lipopolysaccharide, and LY294002 pretreatment reduced p-PI3K, p-Akt and HIF-1α protein expression. Kc2f7 pretreatment reduced the HIF-1α expression (Figures 7G–J), without affecting the expression of p-PI3K and p-Akt. In order to further explore the anti-inflammatory mechanism of propofol in microglia. Insulin was used as an agonist of PI3K and DMOG as a HIF-1α agonist. Insulin pretreatment increased the expression of p-PI3K, p-Akt and HIF-1 α. DMOG pretreatment increased the expression of HIF-1α but had no effect on the expression of p-PI3K and p-Akt. Propofol significantly reduced both insulin and DMOG induced HIF-1α expression (Figures 7K–N). Take together, these results suggested that propofol may regulate multiple targets of the *ROS/PI3K/Akt/mTOR/HIF-1α* signaling pathway.

## 4 Discussion

Our data demonstrated that propofol protects against lipopolysaccharide induced microglia activation, neuroinflammation, neuronal apoptosis and cognitive dysfunction. The underlying mechanisms were associated with the reduction of neuroinflammation and metabolic reprogramming induced by lipopolysaccharide through downregulation of ROS/PI3K/Akt/mTOR/HIF-1α pathway.

Lipopolysaccharide induced systemic inflammation can trigger acutely progressing brain dysfunction and long-term cognitive impairments (Kodali et al., 2021; Savi et al., 2021). However, few studies have focused on the effect of propofol on brain inflammation during endotoxemia or sepsis. A previous study by Huang et al. showed that intravenous administration of propofol for 1 h prior to lipopolysaccharide stimulation prevented microglia activation but not the elevation of TNF-α in the hippocampus (Huang et al., 2019). In the current study, propofol (80 mg/kg, i. p.) not only inhibited lipopolysaccharide (2 mg/kg) induced inflammation in the hippocampus, but also improved cognitive performance.

Recent studies suggested that the metabolic profile changes of immune cells, including brain microglia, are important in regulating their functional activation (Park et al., 2020). In microglia cells, lipopolysaccharide stimuli resulted in an elevation in M1-related pro-inflammatory genes, decreased mitochondrial oxygen consumption (OCR), increased lactate release and extracellular acidification rate (ECAR) (Orihuela et al., 2016; Lu et al., 2022). Therefore, we investigated whether propofol affects the inflammation through the cellular metabolic pathways. We found in this study that propofol significantly inhibited real-time ECAR, an important indicator of glycolysis. The PFKFB3 and HK2 upregulations induced by lipopolysaccharide in microglia were reversed by propofol. Thus, the effects of propofol on cellular metabolic reprogramming may, at least in part, contribute to these cellular signalling anti-neuroinflammatory actions.

Propofol was reported to influence cancer cell metabolism in previous studies. For example, propofol exposure inhibited aerobic glycolysis in HT29 and SW480 colorectal cancer cells *via* inactivation of the NMDAR-CAMKII-ERK pathway (Chen et al., 2018). In addition, propofol suppressed cell carcinogenesis and aerobic glycolysis by decreased GLUT1 expressions in lung cancer cells (Hu et al., 2021). In line with our findings, a recent report showed that propofol suppressed aerobic glycolysis via inhibition of GLUT1-mediated glucose uptake in lipopolysaccharide-activated macrophages (Zeng et al., 2021). However, high concentrations of propofol was found to induce metabolic switch towards glycolysis and cell death in a mitochondrial electron transport chain-dependent manner (Sumi et al., 2018). It remains to investigate how propofol differently affect cellular metabolism in different cell types.

A number of mechanisms have been proposed to account for propofol anti-inflammatory effect in microglia, including downregulation of TLR4 expression (Qin et al., 2013), inactivation of GSK-3β (Gui et al., 2012), inhibition of NMDA receptor and NADPH oxidases (Luo et al., 2013) as well as suppression of miR-155 and miR-221/222 (Zheng et al., 2017; Xiao et al., 2021). Recent study reported that propofol regulated the activity of HIF-1α to reduce prostate cancer cells malignancy (Huang et al., 2014). HIF-1α, one of the key transcriptional regulators of immunity and inflammation, plays a crucial role in regulating microglial cell metabolic reprogramming (Ahn et al., 2012; Ulland et al., 2017; York et al., 2021). In this study, we found that propofol attenuated HIF-1α expression induced by CoCl_2_ and DMOG. The inhibitory effect of propofol on lipopolysaccharide-induced neuroinflammation was no longer exist in the presence of HIF-1α specific inhibitor Kc7f2. Moreover, PI3K/Akt/mTOR signaling pathway acts as an inducer of HIF-1α upstream effectors (Zhang et al., 2021). It has been reported that elevated reactive oxygen species can lead to the activation of PI3K, Akt and mTOR cascades (Chen et al., 2017; Urasaki et al., 2018). Our results further showed that propofol inhibited lipopolysaccharide induced ROS production as well as PI3K, Akt and mTOR phosphorylation. Propofol also significantly reduced insulin induced PI3K signaling activation and HIF-1α expression. All these suggest that ROS mediated PI3K/Akt/mTOR/HIF-1α signaling pathway may be very likely involved in the inhibitory effect of propofol on lipopolysaccharide induced metabolic reprogramming and inflammation.

There are several limitations in the current study that must be acknowledged. First, the propofol acts more as a preventive measure in our experiment design, which may not completely mimic the clinical situation. However, we have previously demonstrated that propofol exerted similar anti-inflammatory effect when it was given 1 hour before or after lipopolysaccharide treatment in microglial cells (Luo et al., 2018). Second, this study demonstrated that propofol significantly reduced lipopolysaccharide-induced elevated expression of caspase-3 in the hippocampus, which supports its potential neuroprotective capability. However, the specific cell type that accounts for increased caspase-3 expression will need to be identified in further study. Third, although we demonstrated propofol inhibited HIF-1α mediated metabolic reprogramming in lipopolysaccharide challenged mice, the involvement of PI3K-Akt-mTOR signaling pathway were not validated in primary microglia *in vivo*. It has been suggested that, in response to lipopolysaccharide, 90% of the genes induced in BV2 cells are also induced in primary microglia (Henn et al., 2009).

In conclusion, we demonstrated that propofol exhibits neuroprotective effects against lipopolysaccharide-induced neuronal injury via the amelioration of neuroinflammation and inhibition of microglial metabolic reprogramming. Propofol is widely used for sedation in mechanically ventilated patients with sepsis. Our finding warrants further preclinical and clinical investigations to explore the therapeutic potential of propofol in sepsis-associated encephalopathy.

## Data Availability

The original contributions presented in the study are included in the article/Supplementary Material, further inquiries can be directed to the corresponding author.
